# A Systematic Review to Assess the Relationship between Disseminated Cerebral Aspergillosis, Leukemias and Lymphomas, and Their Respective Therapeutics

**DOI:** 10.3390/jof8070722

**Published:** 2022-07-11

**Authors:** Brianne N. Sullivan, Mia A. Baggett, Samantha S. O’Connell, Keith M. Pickett, Chad Steele

**Affiliations:** 1 Tulane Brain Institute, School of Science and Engineering, Tulane University, New Orleans, LA 70112, USA; bsulliv7@tulane.edu (B.N.S.); mbaggett1@tulane.edu (M.A.B.); 2 Microbiology and Immunology, School of Medicine, Tulane University, New Orleans, LA 70112, USA; 3 Office of Academic Affairs and Provost, Tulane University, New Orleans, LA 70112, USA; soconne1@tulane.edu; 4 Rudolph Matas Library of the Health Sciences, School of Medicine, Tulane University, New Orleans, LA 70112, USA; kpicket1@tulane.edu

**Keywords:** cerebral aspergillosis, invasive pulmonary aspergillosis, hematologic malignancy, immunosuppression, disseminated disease, mortality

## Abstract

Disseminated disease following invasive pulmonary aspergillosis (IPA) remains a significant contributor to mortality amongst patients with hematologic malignancies (HMs). At the highest risk of mortality are those with disseminated disease to the central nervous system, known as cerebral aspergillosis (CA). However, little is known about the risk factors contributing to disease amongst HM patients. A systematic review using PRISMA guidelines was undertaken to define HM patient subgroups, preventative measures, therapeutic interventions, and outcomes of patients with disseminated CA following IPA. The review resulted in the identification of 761 records, of which 596 articles were screened, with the final inclusion of 47 studies and 76 total patients. From included articles, the proportion of CA was assessed amongst HM patient subgroups. Further, pre-and post-infection characteristics, fungal species, and mortality were evaluated for the total population included and HM patient subgroups. Patients with acute myeloid leukemia and acute lymphoid lymphoma, patients receiving corticosteroids as a part of their HM therapeutic regimen, and anti-fungal prophylaxis constitute the top identified patient populations at risk for disseminated CA. Overall, information presented here indicates that measures for the prevention of IPA should be taken in higher-risk HM patient subgroups. Specifically, the type of anti-fungal therapy used should be carefully considered for those patients with IPA and increased risk for cerebral dissemination. Additional reports detailing patient characteristics are needed to define further the risk of developing disseminated CA from IPA in patients with HMs.

## 1. Introduction

*Aspergillus* (*A.*) species (spp.) are ubiquitous, opportunistic fungal pathogens that, when inhaled, are readily eliminated from the lung of immunocompetent individuals but can lead to the highly lethal infection invasive pulmonary aspergillosis (IPA) in immunocompromised individuals. Specifically, patients undergoing immunosuppressive therapies for stem cell and solid-organ transplants, hematologic malignancies (HM), and the use of immunomodulating drugs such as corticosteroids are at the most significant risk of developing IPA [1,2,3,4]. *A. fumigatus* is the species most frequently attributed to IPA; however, additional species are occasionally identified as the cause of IPA, including *A. niger*, *A. flavus*, and azole-resistant *A. terreus* [5,6,7]. Dissemination of these fungi most frequently occurs via the hematogenous spread of the fungus from the lungs to subsequent organs [8,9,10]. The central nervous system (CNS) is often reported as the most frequent site of *Aspergillus* dissemination from the lung resulting in cerebral aspergillosis (CA); this is particularly true in immunocompromised populations [7,10,11]. In addition to being amongst the most common organs of dissemination, *Aspergillus* infection in the CNS is also regarded as one of the most lethal [12,13,14].

IPA is diagnosed in more than 300,000 immunocompromised patients annually and is associated with a 30–80% mortality rate [2,15,16]. On average, 20–50% of IPA infections result in disseminated disease, with 10–20% reported to result in CA [17,18]. However, this is likely a conservative estimate as the population of CA is greatly under-reported, owning partially to the fact that CA is notably difficult to diagnose, with some cases not being diagnosed until autopsy [19,20,21,22]. Further, as the number of immunosuppressed patients continues to increase, the CA population is likely larger than reported [23,24,25]. Disseminated CA is associated with a particularly poor prognosis, resulting in death in up to 70–100% of patients [23,25,26]. The difficult diagnosis of CA additionally contributes to the high mortality as the symptoms, including fever, headache, mental alteration, or lethargy, etc., are non-specific [5,27]. The difficulty of diagnosis is also due, in part, to the methods which are often invasive and have variable sensitivity and specificity [28]. Latency to diagnose combined with poor therapeutic tools often result in a fatal infection. Within the immunocompromised population, patients with hematologic malignancies (HMs) (i.e., cancers that affect the blood, bone marrow, and lymph nodes) are considered to be one of the most prevalent populations to be diagnosed with disseminated CA [1,29]. HM patient subgroups include various leukemias (acute lymphocytic (ALL), chronic lymphocytic (CLL), acute myeloid (AML), chronic myeloid (CML)), myeloma, and lymphoma (Hodgkin′s and non-Hodgkin′s (NHL)). Additionally, the therapeutics and treatments associated with HMs, such as chemotherapy and stem cell transplants (SCT), leave the patients in a highly immunocompromised state, elevating the risk for opportunistic infections. Given the high proportion of patients reported to have disseminated CA also having HMs, it is essential to identify the most at-risk HM patient subgroups and characteristics for CA so prophylactic measures and therapeutic considerations can be taken.

Up-to-date reports of disseminated CA specifically related to HMs are relatively limited. The risk factors of disseminated CA related to HM patient subgroups and their therapeutics, including cytotoxic drugs, steroids, SCTs, and targeted agents, are also not well documented. This systematic review clarifies the evidence base available around the relationship between HM patients undergoing therapy related to HMs and CA. Additionally, this review aims to identify any relationships between HM patient subgroups and the prevalence of CA, thus, potentially identifying patient subgroups with increased risk. Further, this review aims to identify the post-infection characteristics of CA patients. Lastly, this review addresses any relationships between various patient characteristics, disseminated CA, and mortality. We systematically reviewed the literature using the Preferred Reporting Items for Systematic Reviews and Meta-Analyses (PRISMA) guidelines to address these objectives. A systematic review was performed on the selected study population comparing patients receiving chemotherapy, SCT, corticosteroids, and/or targeted therapies before diagnosis with IPA with disseminated CA. Post-infection characteristics were also compared, including anti-fungal treatment, surgical intervention, *Aspergillus* spp., and mortality. Studies included in this review were published on or before 18 May 2021, and were accessed through four online databases.

## 2. Materials and Methods

The systematic review was conducted according to PRISMA guidelines. The study protocol for this systematic review was registered with the PROSPERO database with the registration number CRD42021288469 [30].

### 2.1. Search Strategy and Study Selections

Data sources used for this systematic review were PubMed/Medline, Embase, Cumulative Index to Nursing and Allied Health Literature (CINAHL) Plus, Web of Science, and GreyLit. All databases were searched from inception to 18 May 2021, and all relevant peer-reviewed studies published were included for systematic review. No limits were placed for language, publication type, etc., in the initial search. The literature search strategy combined all synonyms for the disease “cerebral aspergillosis” combined with “disseminated,” combined with all synonyms for diseases resulting from “hematologic malignancies,” and all synonyms describing “chemotherapy” and related “immunosuppression.” Supplemental Table S1 contains all MeSH terms and keywords that comprise the search strategies used for each database.

All articles retrieved through database searching were imported into Covidence systematic review software (Veritas Health Innovation, Melbourne, Australia), where duplicate records were automatically removed. In Covidence, studies were screened first by title and abstract review and second by full-text review by two independent reviewers (BNS and MAB) in duplicate. Following the first and second reviews, those studies kept and rejected were compared between reviewers, and any discrepancies were resolved by consensus.

### 2.2. Eligibility Criteria

Research articles that met the following defined inclusion criteria were selected for systematic review. Eligible studies needed to include patients with leukemia or lymphoma (any age/gender), diagnosed IPA + CA (proven, any *Aspergillus* spp.) with the primary infection being in the lung and without any concurrent infections by other yeasts, viruses, and/or bacteria, etc., and the outcome of fungal infection. Studies additionally needed to include one or all of the following regarding whether or not patient(s) included in the study were receiving chemotherapy, SCT, and/or another therapeutic for HM. Studies without patients with CA were excluded, and patients without CA in studies included were excluded from the analysis. Study design: any full-text peer-reviewed reports available in English containing original clinical data were considered; this primarily included case reports and series. Further, preprint articles and articles with no full text available were not included. The primary outcomes of interest were mortality and prevalence of CA within HM patient subgroups. Comparison or control groups were not applicable.

### 2.3. Data Abstraction

Data were abstracted independently and in duplicate by two reviewers using standardized data extraction criteria for case reports and studies. The Covidence systematic review software was utilized for data abstraction for case reports. For case series, Google Sheets was used. For case series in which cohort data were additionally available, individual patient data were preferentially used as they provided more-detailed information about underlying HMs, treatment, and outcomes. Two independent investigators (BNS, MAB) abstracted the following data, when available, from eligible articles: general study information (including title, authors, PMID, country study was conducted in, year of infection diagnosis, and year of publication), study characteristics (case study versus series), participant characteristics (including age, gender, type of leukemia or lymphoma, neutropenic status, absolute neutrophil count (ANC) or white blood cell count (WBC), and additional sites of dissemination if any), information about the interventions (including chemotherapy regimen, SCT, any non-cytotoxic therapeutics pre- or post-IPA, prophylactic anti-fungal regimens, therapeutic interventions for aspergillosis, and surgical interventions), type of *Aspergillus*, and outcome measures (survival). Abstracted data were compared between the two reviewers, and any discrepancies were resolved by consensus. Upon resolving discrepancies, data were synthesized into a single form that was maintained on Google Sheets.

### 2.4. Assessment of Study Quality

The included publications were assessed for risk of bias for selection, ascertainment, causality, and reporting based on the modified Pearson Case Report Quality scale proposed by Murad and colleagues [31]. For each bias domain, levels of bias were rated as high, low, or unclear, based upon the response of no, yes, or unclear, respectively, to the prompting questions. The overall risk of bias of a study was deemed low if the study had a low risk of bias for all domains. The overall risk of bias was considered unclear if a study had an unclear risk of bias for at least one domain. Lastly, the overall risk of bias was deemed high if a study had a high risk of bias for at least one domain. All responses were recorded through Covidence systematic review software (Supplemental Table S2). The consensus of quality was reached by two independent researchers (BNS, MAB) for each study.

### 2.5. Data Synthesis and Analysis

A narrative summary approach was used to detail the key study characteristics and systematic review findings. As each study represented an individual patient or patients, data were synthesized and described in this way. The data were pooled to determine the prevalence of underlying HM patient subgroups, treatments, outcome, and other pertinent variables in the patient population. In some analyses, studies were excluded if relevant data were not available. For this reason, the number of patients varies in each analysis. Further, due to heterogeneity in study design, statistical analysis of the data collected from the 47 studies was not undertaken.

## 3. Results

### 3.1. Search Results

**Figure 1:**
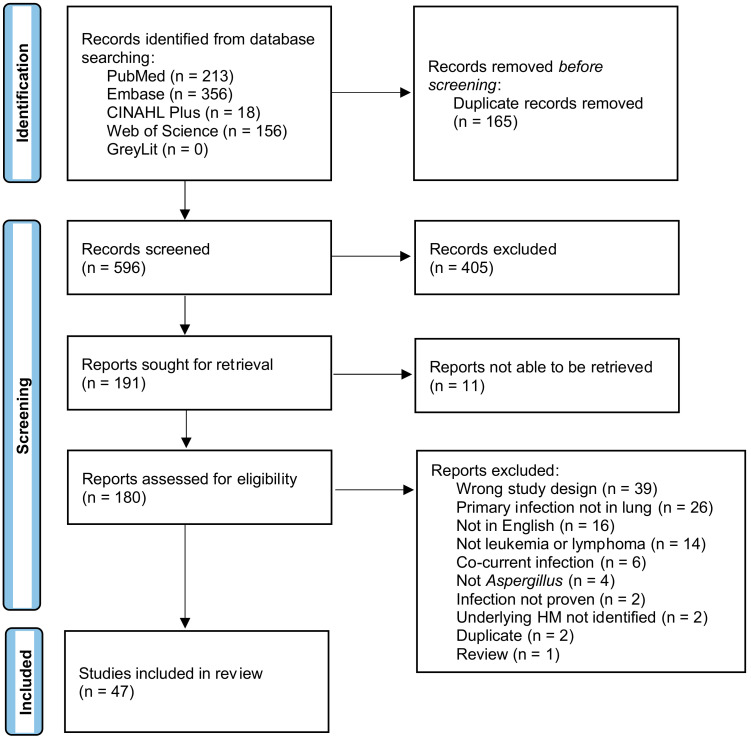
Prisma flow chart.

The PRISMA flow diagram [32] detailing the search results is shown in Figure 1. A search of PubMed, Embase, CINAHL, Web of Science, and GreyLit was conducted and yielded 761 records. After removing 165 duplicates by Covidence, 596 records were screened for title and abstract. This initial screening resulted in 191 records being sought for retrieval, 180 reports were assessed for eligibility, and 11 were unable to be retrieved. A total of 133 records were deemed ineligible and were excluded. Studies were excluded for the following reasons: the primary infection was not in the lung, there was no infection in the brain, the manuscript was not available in English, the patients did not have an HM prior to infection, or the HM patient subgroup was not specified/proven, the infection was not *Aspergillus* or was not proven to be, patients had a co-current infection with another yeast, bacteria, virus, etc., the study was a duplicate that Covidence did not remove in the initial screening, the article was a review, and/or the study was the wrong design where no relevant data could be extracted. Many studies met multiple criteria for exclusion but were only tagged with one exclusion criterion. The remaining 47 case reports, case series, and observational studies, all of which contained one or more patients with IPA + CA, underwent data extraction and were included in the final systematic review. Seventy-six HM patients with CA disseminated from IPA were included from the 47 studies, summarized in Table 1.

### 3.2. Quality Appraisal

Each study′s quality was assessed and detailed in Supplementary Table S2. The overall outcome of the quality appraisal is synthesized in Supplementary Scheme S1. For evaluating the selection bias of patients included, it was assessed whether the patient(s) included in the case report or series represented the whole experience of the investigator. Overall, there was a relatively low risk of selection bias, with 97.87% of studies being deemed as having a low risk of bias and 2.13% with an unclear risk of bias. There was a low risk of ascertainment bias for evaluating the exposure and outcome of each study, with 100% of studies for both criteria being considered as having a low risk of ascertainment bias. Each study was evaluated to determine if other alternative causes that may explain the observation were ruled out when assessing causality bias. Further, 55.32% of studies had a low risk of causality bias, 31.91% had an unclear risk of bias, and 12.77% had a high risk of causality bias. Causality bias was further ascertained by evaluating the follow-up time to determine the outcome, and 97.87% of studies were found to have a relatively low risk of causality bias, and 2.13% with an unclear risk of bias. Overall, the causality bias was moderate, with 76.60% of studies having low risk of causality bias, 17.02% with unclear risk of bias, and 6.38% of studies with a high risk of causality bias. Finally, the risk of reporting bias was determined by evaluating whether the case(s) were described with enough detail for researchers and/or practitioners to replicate to make inferences related to their practice. Here, 63.83% of studies were found to have low risk of reporting bias, 27.66% have unclear risk of bias, and 8.51% have a high risk of bias. Essentially, most case reports and studies had a low risk of bias, with 65.96% with low risk, 31.91% with an unclear risk of bias, and 2.13% having a high risk of bias.

### 3.3. Demographic Characteristics

The demographic characteristics of all patients included are detailed in Table 2. Reported gender amongst all patients is approximately half male-identifying (54.17%, *n* = 39) and half female-identifying (45.83%, *n* = 33), the gender of four patients was not reported. The age (*n* = 73) of patients ranged from 0.5 to 87 years (mean = 32.5; SD = 21.9). The number of children (<18 years), young adults (18–49 years), and older adults (≥50 years) was relatively similar between all groups.

### 3.4. Underlying Disease

The details regarding the HM patient subgroup reported amongst all patients are included in Table 3. AML was the most frequently reported HM patient subgroup followed closely by ALL. Reported less frequently amongst the population included herein were NHL and CLL. For CML and myeloma, only one patient was reported for each HM amongst the patient population. No cases of disseminated CA included were reported in patients with Hodgkin’s lymphoma.

### 3.5. Prevalence of Known Risk Factors for IPA in the IPA + CA Population

Prior to onset of infection, neutropenia, a common risk factor, was identified in 40 out of the 51 patients where data were available regarding WBC or ANC levels (Table 3). Herein, neutropenia was defined as an ANC or WBC ≤ 1500 cells/μL. Individual patient data regarding WBC or ANC are detailed in Supplemental Table S3. All patients that were considered as neutropenic, except for one, were also receiving chemotherapy related to their underlying HM. Within HM patient subgroups, at least 75% of patients within each group were identified as being neutropenic, apart from CLL in which only 50% of patients with data available were neutropenic.

Chemotherapy was given to 88.06% (*n* = 59) of patients prior to infection (Table 3). In patients with data available regarding the chemotherapy regimen (*n* = 27), the most prevalent chemotherapy given was cytarabine (*n* = 18). Cytarabine was most frequently given in combination with one or more other chemotherapies (88.89%), such as daunorubicin/doxorubicin (*n* = 7), etoposide (*n* = 5), vincristine (*n* = 4), methotrexate (*n* = 4), fludarabine (*n* = 4), and idarubicin (*n* = 4). Most patients receiving chemotherapy had at least two or three (*n* = 7) chemotherapeutic agents in their therapeutic regimen. In patients in which the stage of chemotherapy was reported, 73.91% (*n* = 17) were in the induction phase, with the remaining 26.09% (*n* = 6) in the consolidation phase. In most HM patient subgroups, at least 75% of patients within each were receiving chemotherapy, except for CLL patients, in which only 37.5% (*n* = 3) of patients received chemotherapy at the time of infection (Table 4).

Several targeted, non-chemotherapeutic, anti-cancer therapies were reported. Most notable is ibrutinib (*n* = 11), which was included in the therapeutic regimen for 87.50% of CLL (*n* = 7) and 36.36% of NHL (*n* = 4) patients and was frequently given with rituximab (*n* = 6), and/or corticosteroids (*n* = 8). In patients who received ibrutinib, 45.45% (*n* = 5) were not on chemotherapy, and only 18.18% (*n* = 2) of patients on ibrutinib were on prophylactic antifungal. Overall, 45.45% (*n* = 5) succumbed to infection, and 88.89% of patients *Aspergillus spp.* identified were found to have *A*. *fumigatus*.

Less frequently reported were other targeted therapies such as venetoclax (*n* = 1), immunotherapies such as obinutuzumab (*n* = 2), immunoglobulin (*n* = 4), interleukin-2 (IL-2) (*n* = 2), alemtuzumab (*n* = 1), anti-CD52 (*n* = 1), and biologics such as granulocyte-colony stimulating factor (G-CSF) (*n* = 2) and L-asparaginase (*n* = 7).

Prior to infection, SCTs were done in 36.21% (*n* = 21) of the population. In the cases in which the type of SCT was identified (*n* = 14), the majority were allogenic (78.57%, *n* = 11) as compared to autologous (21.43%, *n* = 3) (Table 3). In groups with more than one patient, NHL patients had the highest prevalence of SCT prior to infection (54.55%, *n* = 6), and ALL patients had the least (12%, *n* = 3) (Table 4).

At the time of infection, 61.11% (*n* = 33) of patients were taking corticosteroids (Table 3). In the patient population in which the type of steroid prescribed was identified (*n* = 21), the most prevalent corticosteroid taken was prednisone (61.90%; *n* = 13), followed by dexamethasone (38.10%; *n* = 8). In most HM patient subgroups, approximately half of the patients were receiving corticosteroids prior to infection, apart from AML patients, of which 30% were being given corticosteroids at the time of infection (Table 4).

To determine if there was a combinatorial effect of risk factors relative to the incidence of cerebral dissemination, the presence and/or absence of multiple variables was evaluated (Table 5). Corticosteroids and chemotherapy together were the most prevalent combination of risk factors, with approximately half of patents with reported data receiving both prior to infection. Less prevalent was SCT combined with chemotherapy or corticosteroids prior to infection as only about one-quarter of patients were reported to have received those therapies prior to infection.

### 3.6. Prophylactic Anti-Fungal Treatment

Data were available regarding the prescription of anti-fungal prophylaxis for 48 patients, of which 47.91% (*n* = 23) were taking anti-fungal drugs at the time of fungal infection (Table 3). Details for the type and regimen of anti-fungal were available for 13 patients. Amphotericin B (AmB) was the most prevalent anti-fungal drug used, with it being prescribed for 69.23% (*n* = 9) of patients before fungal infection. The second most prevalent was fluconazole, accounting for about 30.77% (*n* = 4) of patients given prophylactic anti-fungal drugs. Itraconazole was given to one patient (7.69%). All anti-fungal prophylactics were primarily given singularly, with only one patient receiving AmB + fluconazole in combination [65].

Further, the prevalence of anti-fungal prophylaxis in patients receiving immunosuppressive therapies before fungal infection was evaluated (Table 6). Approximately 45% of patients undergoing chemotherapy received anti-fungal prophylaxis. In patients who were given corticosteroids or who received SCT prior to infection, about one-quarter of those patients also received anti-fungal drugs preceding the invasive fungal infection (IFI).

The prevalence of anti-fungal prophylaxis in most HM sub-populations ranged from approximately 25–35%, except for NHL patients, in which more than 50% were receiving anti-fungal prophylaxis at the time of infection (Table 4).

### 3.7. Treatment

Following the diagnosis of proven infection, 91.30% (*n* = 66) of patients were given antifungal therapy (Table 7). In cases where the specific anti-fungal(s) used were detailed (*n* = 61), the top anti-fungal therapy given, either alone or in combination with additional therapeutics, was AmB, with 80.33% (*n* = 49) of patients receiving it as part of their regimen. Liposomal AmB (L-AmB) was given to 19.67% (*n* = 12) of patients. Additionally, 22.95% (*n* = 14) of patients were given AmB singularly. When given in combination with additional anti-fungals, AmB was most frequently given with voriconazole alone (16.39%, *n* = 10) or in combination with other antifungals (18.03%, *n* = 11). AmB was given with fluconazole in 14.75% (*n* = 9) or with itraconazole in 8.19% (*n* = 5) of patients. The second most reported anti-fungal therapy prescribed to patients was voriconazole, accounting for 49.18% (*n* = 30) of patients, most often included as a part of a therapeutic regimen. Voriconazole was often given with caspofungin with (8.19%, *n* = 5) or without AmB (4.92%, *n* = 3). Posaconazole was included in the treatment regimen of several patients (6.56%, *n* = 4), and was given along with voriconazole for all patients, with AmB for 3/4 and caspofungin for 2/4. Other anti-fungal drugs, including echinocandin (*n* = 2), isavuconazole (*n* = 2), micafungin (*n* = 2), natamycin (*n* = 1), and fluconazole (*n* = 1), were occasionally included in patients’ therapeutic regimens, albeit less frequently than others mentioned above.

Of the 58 patients with data regarding surgical interventions following diagnosis, 44.83% (*n* = 26) of patients underwent surgical intervention for the IFI (Table 7).

### 3.8. Species

Among the 39 patients in which *Aspergillus* isolates were identified to the species level, the most frequent species identified was *A. fumigatus* (64.10%, *n* = 25), followed by *A. flavus* (25.64%, *n* = 10). Single cases of *A. terreus*, *A. felis*, and *A. niger* were identified amongst the patient population included (Table 8). Two patients were found to have two species of *Aspergillus* identified. The first patient was a 12-year-old with ALL who had a co-infection by *A. fumigatus* and *A. flavus*; the patient survived the infection [36]. The second patient was an 18-month-old with ALL who had co-infection by *A. fumigatus* and *A. niger*; the patient did not survive [28].

### 3.9. Mortality

Overall, ~54% (*n* = 41) of patients included succumbed to CA (Table 9). Evaluation of the overall mortality in each HM patient subgroup demonstrated patients with acute lymphomas (AML, ALL) succumbed to infection at a rate of 50% (*n* = 15) and 48% (*n* = 12), respectively. The mortality rate was much higher in the NHL population with 90.91% (*n* = 10) of patients with NHL succumbing to the IFI.

Examining mortality according to pre-infection risk factors showed patients exposed to chemotherapy or corticosteroids before infection to have a mortality rate of 59.32% (*n* = 35) and 60.61% (*n* = 20), respectively. In patients that did not receive corticosteroids before infection, the mortality rate was lower, with 33.33% (*n* = 7) of patients succumbing to infection. In patients that underwent SCT before IFI, 76.19% (*n* = 16) died due to the infection (Table 9).

For patients that received anti-fungal therapy, 51.52% (*n* = 34) succumbed to infection, while 100% (*n* = 3) of patients that did not receive anti-fungal treatment died due to infection (Table 9).

Patients that underwent surgical intervention had a mortality rate of 34.62% (*n* = 9) associated with the infection. Conversely, patients who did not undergo surgical intervention had higher mortality rates, with 62.50% (*n* = 20) of patients succumbing to infection. When the surgical intervention was combined with anti-fungal therapy, the mortality rate was 36% (*n* = 9) (Table 9).

The *Aspergillus* spp. associated with the highest mortality rate was *A. flavus*, with 90.00% (*n* = 9) of patients infected by that species succumbing to infection (Table 8). The most-reported species identified in patients, *A. fumigatus*, was associated with a 47.62% (*n* = 10) mortality rate.

## 4. Discussion

To our knowledge, this is the first comprehensive systematic review of the literature focusing on disseminated CA following IPA in patients with HMs. In this study, we examined the characteristics of a large number of disseminated CA cases following IPA in HM patients published as single case reports, case series, or as a part of larger observational studies. All cases included had a proven *Aspergillus* spp. infection. The mortality rate due to CA was 53.95% overall for patients included in this systematic review.

Like studies focusing on IPA in patients with HMs, we found the predominant HM patient subgroup diagnosed with disseminated CA to be AML, closely followed by ALL [78,79,80,81]. Interestingly, estimates of the global incidence and prevalence of HMs have demonstrated the top reported HM patient subgroups to be NHL and CLL; however, in studies of IPA, and disclosed within this systematic review, have demonstrated those to be of mid-level prevalence. Conversely, the most prevalent HM patient subgroups for IPA and disseminated CA, AML and ALL, are globally regarded as mid-to low-level prevalence [82,83]. This suggests that the high prevalence of AML and ALL patients diagnosed with IPA or IPA coupled with disseminated CA is potentially due, at least in part, to the anti-cancer therapeutic regimen(s) given to those patients. In fact, several reports have linked the prevalence of IFIs and the chemotherapeutic regimens used in the acute leukemia populations [84,85]. Indeed, the top two cytotoxic drugs reported in the cases included in this systematic review were cytarabine and daunorubicin, longstanding chemotherapeutics for acute leukemia. However, in our systematic review of the literature, we did not find anti-fungal prophylaxis to be more prevalent in the acute leukemia population compared to other HM patient subgroups; rather, it was less than other populations, like those with NHL (Table 4). Altogether, more aggressive monitoring prevention, and implementation of anti-fungal drugs into the therapeutic regimen of HM patients, particularly those with acute leukemias, is likely required for the prevention and/or reduction of the highly fatal CA.

Immunosuppression related to the treatment of HMs has long been considered a primary risk factor for IPA. Historically, immunosuppression in patients with HMs has been related to (i) prolonged neutropenia, primarily resulting from the use of chemotherapeutic agents; (ii) immunosuppressive drugs for the prevention and/or treatment of graft versus host disease (GvHD) following allogeneic hematopoietic-SCT; and (iii) corticosteroids prescribed for a range of indications during cancer care, including the reduction of chemotherapy side-effects, anticancer effects, and as a non-specific immunosuppressant following SCT. On average, 50–90% of IPA patients with underlying HMs received chemotherapy before infection [79,86]. Likewise, 88.06% and 78.43% of IPA patients with disseminated CA patients included in this systematic review of the literature received chemotherapeutic agents and were neutropenic, respectively, prior to infection. SCT is conducted in about 20–35% of HM patients with IPA, with allogenic being more prevalent than autologous [18,79,80]. In the population of HM patients with disseminated CA following IPA, similar results were found, with about 36% of patients receiving SCT prior to infection, most of whom received allogenic SCTs. In studies of IPA, approximately 25–45% of patients with underlying HMs were reported to be receiving corticosteroids at the time of infection [18,80]. Interestingly, greater than 60% of the CA patients included herein were prescribed corticosteroids at the time of infection. The elevated prevalence of corticosteroids in HM patients with CA disseminated from IPA compared to HM patients with IPA alone points to a potential factor that increased the susceptibility of developing disseminated CA. Although, it is difficult to draw definitive conclusions related to specific treatments and their impact on developing CA as there is no way to account for all potential variables. The data presented here indicate that HM patients with corticosteroids included in their anti-cancer therapy should be closely monitored and receive prophylactic anti-fungal drugs to prevent the development of this severe disease.

Recently, targeted anti-cancer therapies have become more frequently attributed to increased risk of IFIs, including IPA [87]. One of the most prominent targeted therapies is ibrutinib, a bruton tyrosine kinase (BTK) inhibitor, primarily prescribed to CLL and NHL patients. Ibrutinib is used as a single-agent therapy or as a part of combination therapy with other anti-cancer drugs such as rituximab, an anti-CD20 monoclonal antibody. Although CLL and NHL have not historically been considered as high-risk for developing IPA, the addition of ibrutinib and/or rituximab has been associated with increased prevalence of IPA in these patients [88]. Herein, we report that 85.70% of CLL and 36.36% of NHL patients were given ibrutinib, frequently given in combination with rituximab and/or corticosteroids. Most patients receiving ibrutinib at the time of infection had received chemotherapy prior to initiating ibrutinib [33,42,45,56,71,75]. Only two patients were treatment naïve prior to ibrutinib therapy, and one patient began ibrutinib co-currently with chemotherapy [33,42,72]. Recently, there have been several reports of CA in patients receiving ibrutinib [89,90,91,92,93,94]. While the number of reports at this time is relatively small, it bears noting as historically, the number of CLL patients diagnosed with CA has been relatively low in comparison to patients with other HMs. Thus, this indicates the importance of investigating the incidence of CA in patients receiving ibrutinib therapy to potentially identify an at-risk population.

Other targeted therapies, immunotherapies, and biologics given to HM patients prior to fungal infection, such as L-asparaginase, immunoglobulins, and venetoclax, among others, were administered to only a few patients, which does not permit us to make any inferences regarding their influence for the susceptibility for disseminated CA in this population. However, given the mechanisms of action of some of these drugs and previously published reports, it is reasonable that they could have impacted the immune status of patients [87,95,96]. More reports on non-chemotherapeutic drugs given to HM patients are required to draw any substantial conclusions.

Anti-fungal prophylaxis has become a common addition to the treatment regimen of HM patients, with and without SCTs [97,98,99]. The addition of anti-fungal prophylaxis is thought to contribute to the overall reduction of IPA cases amongst immunocompromised individuals [97,98]. Further, anti-fungal prophylaxis in HM patients is a positive predictor of survival in breakthrough cases of IPA [97,100]. The number of HM patients prescribed antifungal drugs prophylactically typically ranges from ~15–45% [101,102]. It should be noted, however, that the prevalence of antifungal prophylaxis in HM patients is on the rise with the development of new antifungal drugs and repeated demonstration of the efficacy of using these drugs prophylactically [86,103,104]. Here, we report that 47.91% of HM patients had breakthrough IPA with disseminated CA. Although, data for this were only retrievable from two-thirds of the studies, which is likely attributable to the age of some studies and the lack of antifungal prophylaxis.

Posaconazole is a prophylactic antifungal that has been consistently found to be the most effective at preventing IPA, with as little as 1% of neutropenic patients on prophylactic posaconazole with breakthrough IPA [86,97]. No patients included in this systematic review received posaconazole prophylactically. Instead, the majority of patients received AmB, followed by fluconazole (Table 3), both of which have been found to be less efficacious in preventing IPA, notably fluconazole [86]. However, it is unknown whether the infection was due to wrong anti-fungal drug choice, insufficient drug levels in the CNS or host, or fungus-specific issues and thus warrants further investigation.

Historically, AmB has been considered as the standard of care for patients with IPA. However, one study compared voriconazole with AmB as primary treatment for IPA infections and overall exhibited improved survival and response rates [105]. Improved response with voriconazole was further demonstrated through higher successful outcomes in HM patients, patients with extrapulmonary involvement, and others suggesting voriconazole to be superior to AmB at ameliorating *Aspergillus* driven infections. Further still, treatment with voriconazole resulted in significantly fewer adverse events. Another study examining the inclusion or exclusion of voriconazole in the treatment of IPA in HM patients found the overall mortality of those receiving voriconazole to be 5%, significantly lower than the 49% mortality rate associated without voriconazole [100]. By and large, in the population of disseminated CA disclosed herein, AmB was the number one therapeutic prescribed, whether singularly or in combination. The second most prescribed in the patients included within this systematic review was voriconazole, which was often given in combination with AmB and/or other anti-fungal drugs such as caspofungin and posaconazole. Generally, the inclusion of voriconazole reduced the overall mortality of disseminated CA. The inclusion of voriconazole with or without AmB was associated with ~30% mortality, while AmB in the absence of voriconazole was associated with ~75% mortality. While it is difficult to draw conclusions due to the inability to exclude confounding factors, the reduction of mortality associated with voriconazole suggests its therapeutic potential for CA and warrants further investigation.

In agreement with previously published cases of IPA in HM patients, *A. fumigatus* was the most common isolate identified in this systematic review [80,81,106,107]. Here, *A. flavus* was the second most common isolate identified in HM patients with IPA disseminated to CA. However, the trends observed in reports of HM patients with IPA are inconsistent, with some reporting *A. flavus* or *A. terreus* as the second most common isolate identified in HM patients with IPA [18,80,100,106]. In the cases included in this systematic review, only one report of infection by *A. terreus* was identified. Of note, while the population of *A. flavus* was less than half of the *A. fumigatus* population, infection by *A. flavus* was associated with a 90% mortality, approximately double that of *A. fumigatus* (Table 9).

### Limitations

One of the primary limitations of the study was missing data. While most single case series provided adequate detail about patient history, treatment regimens, and outcome, this was not always the case for the case series and observational studies. Additionally, while many studies detailed whether, for example, chemotherapy and corticosteroids were included in patient treatment regimens, details on the type, dosing, and duration were often excluded. More to this point, disclosure of ANC levels was frequently neglected, despite neutropenia being a well-established risk factor for infection that is often resultant from chemotherapy. Further, several studies included were published over a decade ago, and thus missing data could not be retrieved. A limitation of the wide range of dates in which the studies were conducted is that therapeutic standards have changed vastly with advancements in modern medicine, thus often making it difficult to make direct or meaningful comparisons. An additional limitation pertaining to the range of dates of the studies included is that the tools and criteria for diagnosing proven *Aspergillus* infection have changed throughout time, thus we had to rely on standards appropriate for the time of diagnosis and best judgment to determine whether a case met our stringent criteria for inclusion. In doing so, it is possible that articles were excluded or included when others would not have made that judgment, thus introducing potential bias.

Further, during the screening process, many studies with CA patients had to be excluded because they did not provide adequate information about patients included with CA. Rather, the characteristics provided were for all patients, and thus no population data specific to CA could be retrieved from those articles. For this reason, single case reports, case series, and observational studies in which individual data could be retrieved were preferentially used. Ultimately, the lack of patient data at that level severely limited the number and types of articles that could be included, and thus it is possible some important information was excluded. Further, as we were unable to retrieve CA cohort data from larger studies investigating aspergillosis, a meta-analysis was not able to be conducted. Due to this, we were unable to potentially identify distinguishing factors amongst patient cohorts with disseminated CA that may have provided critical information to better identify high-risk populations. Therefore, for improved analysis of this population and potential identification of critical risk factors, more articles are required that distinguish and detail cohort characteristics for those with disseminated CA in the invasive aspergillosis population.

## 5. Conclusions

Disseminated cerebral aspergillosis poses a significant risk to the immunocompromised population as it is associated with a high mortality rate. Historically one of the most at-risk populations for IPA has been those with HMs, which is often attributed to the neutropenia associated with chemotherapy. With the brain being amongst the top sites of dissemination and the subsequent disease being associated with a high mortality rate, delineating the HM populations and characteristics associated with CA posed a critical need. Despite the limitations, this systematic review provides a comprehensive evidence base and analysis of a large population of HM patients with IPA with disseminated CA. Overall, the outcome of this systematic review highlights the need for more stringent incorporation of anti-fungal drugs in high-risk HM patient subgroups such as those with acute leukemias receiving chemotherapy and/or corticosteroids to reduce the incidence and mortality of this highly deadly disease.

## Figures and Tables

**Table 1:** Characteristics of included studies.

Ref.	Country of Diagnosis	Year of Diagnosis	Patient #	Gender	Age (Years)	HM	*Aspergillus* Species	Outcome
[33]	France	2017	1	M	75	CLL	*A. fumigatus*	Survived
			2	M	65	CLL	*A. fumigatus*	Survived
[34]	Germany	1992	3	M	17	ALL	*Aspergillus* spp.	Survived
			4	M	16	ALL	*A. fumigatus*	Died
[35]	Spain	2011–2017	5	M	58	AML	*Aspergillus* spp.	Died
			6	M	52	AML	*Aspergillus* spp.	Died
			7	M	56	MM	*Aspergillus* spp.	Died
[36]	Germany	2002	8	F	12	ALL	*A. fumigatus, A. flavus*	Survived
			9	F	63	AML	*A. fumigatus*	Died
		2003	10	F	4	ALL	*A. fumigatus*	Survived
[37]	USA	1985–1990	11	NA	NA	NHL	*A. flavus*	Died
			12	NA	NA	NHL	*A. flavus*	Died
			13	NA	NA	NHL	*A. flavus*	Died
[38]	USA	1985–1994	14	F	36	NHL	*A. flavus*	Died
			15	M	38	NHL	*A. flavus*	Died
			16	M	16	ALL	*A. fumigatus*	Died
[39]	USA	1997–1999	17	M	16	ALL	*Aspergillus* spp.	Died
			18	M	6	AML	*Aspergillus* spp.	Survived
[40]	Japan	1995	19	F	71	AML	*Aspergillus* spp.	Died
		1978–1995	20	F	71	AML	*Aspergillus* spp.	Died
			21	F	57	ALL	*Aspergillus* spp.	Died
[41]	Germany	1988	22	M	49	ALL	*Aspergillus* spp.	Survived
		1989	23	F	23	AML	*Aspergillus* spp.	Died
[42]	USA	2014–2016	24	M	65	NHL	*A. fumigatus*	Died
			25	F	87	NHL	*A. fumigatus*	Died
			26	M	49	NHL	*A. fumigatus*	Survived
[43]	USA	2001	27	F	6	AML	*Aspergillus* spp.	Survived
			28	M	6	AML	*Aspergillus* spp.	Survived
[44]	Italy	2015	29	F	65	AML	*Aspergillus* spp.	Died
			30	F	60	AML	*Aspergillus* spp.	Survived
[45]	Unknown	2014–2017	31	M	67	CLL	*Aspergillus* spp.	Died
			32	M	71	CLL	*Aspergillus* spp.	Died
[46]	Netherlands	2019	33	F	18	ALL	*A. fumigatus*	Survived
			34	F	15	ALL	*A. fumigatus*	Survived
[47]	USA	1982–1990	35	F	22	AML	*A. flavus*	Died
			36	F	31	ALL	*Aspergillus* spp.	Died
			37	F	57	AML	*Aspergillus* spp.	Died
			38	F	32	ALL	*Aspergillus* spp.	Died
			39	F	20	AML	*A. flavus*	Died
			40	F	21	ALL	*Aspergillus* spp.	Died
[48]	Netherlands	2007–2009	41	F	13	NHL	*A. fumigatus*	Died
		2007–2010	42	M	60	AML	*A. fumigatus*	Survived
[29]	USA	1956–1985	43	M	60	AML	*Aspergillus* spp.	Survived
			44	F	62	AML	*Aspergillus* spp.	Survived
			45	M	59	NHL	*Aspergillus* spp.	Died
			46	M	14	ALL	*Aspergillus* spp.	Died
[49]	USA	1995–2002	47	NA	10	AML	*A. flavus*	Died
[50]	Taiwan	1987–2005	48	M	11	AML	*Aspergillus* spp.	Survived
[51]	Netherlands	2007	49	F	16	ALL	*A. fumigatus*	Survived
[52]	United Kingdom	2006	50	M	34	AML	*A. fumigatus*	Survived
[53]	USA	1991	51	F	6	ALL	*A. fumigatus*	Survived
[54]	USA	1981	52	M	23	AML	*A. terreus*	Died
[55]	France	1994–1995	53	M	61	AML	*A. fumigatus*	Died
[56]	Israel	2018	54	M	37	NHL	*A. fumigatus*	Died
[57]	Spain	1997	55	M	43	ALL	*Aspergillus* spp.	Died
[58]	Japan	2017	56	M	15	AML	*Aspergillus* spp.	Died
[59]	India	2011	57	M	14	ALL	*Aspergillus* spp.	Died
[60]	Thailand	1991–2000	58	F	36	ALL	*A. fumigatus*	Died
[61]	China	2012	59	M	53	AML	*Aspergillus* spp.	Survived
[62]	USA	Before 1987	60	F	32	AML	*Aspergillus* spp.	Survived
[63]	Japan	1995	61	M	41	AML	*A. flavus*	Died
[64]	Italy	2000	62	F	53	CLL	*A. flavus*	Survived
[65]	Germany	1996	63	F	62	AML	*Aspergillus* spp.	Survived
[66]	Germany	2003	64	F	9	AML	*A. fumigatus*	Survived
[67]	Korea	2011	65	F	31	AML	*Aspergillus* spp.	Survived
[68]	Germany	1980	66	M	12	ALL	*A. fumigatus*	Survived
[69]	France	1999	67	F	30	CML	*A. fumigatus*	Died
[70]	USA	2009	68	M	17	ALL	*Aspergillus* spp.	Survived
[71]	USA	2015	69	M	76	CLL	*A. fumigatus*	Survived
[72]	USA	2017	70	M	62	CLL	*A. fumigatus*	Survived
[73]	Italy	2017	71	F	3	ALL	*Aspergillus* spp.	Survived
[74]	France	2002	72	M	57	AML	*Aspergillus* spp.	Survived
[75]	Australia	2018	73	M	66	CLL	*A. felis*	Survived
[76]	Greece	2004	74	M	2	ALL	*A. fumigatus*	Survived
[28]	Iran	2018	75	M	1.5	ALL	*A. fumigatus, A. niger*	Died
[77]	Italy	2015	76	F	0.5	ALL	*Aspergillus* spp.	Survived

Abbreviations: Ref. = reference; M = Male; F = Female; NA = Data not available; AML = Acute myeloid leukemia; ALL = Acute lymphocytic leukemia; CML = Chronic myeloid leukemia; CLL = Chronic lymphocytic leukemia; NHL = Non-Hodgkin’s lymphoma; MM = Multiple myeloma; *A*. = *Aspergillus*; spp. = *Aspergillus* species.

**Table 2:** Demographic characteristics of all included studies.

Age of all Patients (*N ^a^* = 73) (Mean ± SD (Years))	32.5 ± 21.9
	**% (n ^b^)**
*<18 years*	34.25% (25)
≥*18, <50 years*	27.40% (20)
≥*50 years*	38.36% (28)
Identify as male (*N* = 72)	54.17% (39)

^a^ N = total number of patients with available data for characteristic. ^b^ n = number of patients with defined characteristic within group. % = # with defined characteristic/# with available data for characteristic. Abbreviations: SD = standard deviation.

**Table 3:** Patient characteristics.

	% (n ^a^)
**Underlying HM patient subgroup**	(*N ^b^* = 76)
AML	39.47% (30)
ALL	32.89% (25)
CML	1.32% (1)
CLL	10.53% (8)
NHL	14.47% (11)
MM	1.32% (1)
**Neutropenic** (*N = 51*)	78.43% (40)
**Immunosuppressive therapies**	
**Chemotherapy** (*N* = 67)	88.06% (59)
***Phase*** (*N* = 23)	
Induction	73.91% (17)
Consolidation	26.09% (6)
***Type + regimen*** (*N* = 27)	
Mono therapy	18.52% (5)
Multi therapy	81.48% (22)
Regimen includes Cytarabine	66.66% (18)
Regimen includes Daunorubicin	40.74% (11)
Regimen includes Vincristine	33.33% (9)
**SCT** (*N* = 58)	36.21% (21)
Allogenic	78.57% (11)
Autologous	21.43% (3)
**Corticosteroids** (*N* = 54)	61.11% (33)
***Type*** (*N* = 21)	
Prednisone	61.90% (13)
Dexamethasone	38.10% (8)
**Prophylactic anti-fungal**	
**Yes** (*N* = 48)	47.91% (23)
***Type + Regimen*** (*N* = 13)	
AmB^c^	61.54% (8)
Fluconazole	23.08% (3)
Itraconazole	7.69% (1)
AmB + fluconazole	7.69% (1)

^a^ n = number of patients with defined characteristic within group. ^b^ N = total number of patients with available data for characteristic. ^c^ 1 patient received broad-spectrum anti-microbials in addition to AmB. % = # with defined characteristic/# with available data for characteristic. Abbreviations: HM = Hematologic malignancy AML = Acute myeloid leukemia; ALL = Acute lymphocytic leukemia; CML = Chronic myeloid leukemia; CLL = Chronic lymphocytic leukemia; NHL = Non-Hodgkin lymphoma; MM = Multiple myeloma; SCT = Stem cell transplant; AmB = Amphotericin B.

**Table 4:** Risk factors for disease per HM patient subgroup.

	Chemotherapy	Corticosteroids	SCT	Anti-Fungal Prophylaxis
**HM**	(*N ^a^* = 59)	(*N* = 33)	(*N* = 21)	(*N* = 23)
**% (n ^b^)**				
**AML** (*N* = 30)	73.33% (22)	30% (10)	30% (10)	23.33% (7)
**ALL** (*N* = 25)	88% (22)	48% (12)	12% (3)	28% (7)
**CML** (*N* = 1)	100% (1)	100% (1)	100% (1)	-
**CLL** (*N* = 8)	37.5% (3)	50% (4)	-	33.33% (2)
**NHL** (*N* = 11)	90.91% (10)	45.45% (5)	54.55% (6)	54.55% (6)
**MM** (*N* = 1)	100% (1)	100% (1)	100% (1)	100% (1)

^a^ N = total number of patients with characteristic. ^b^ n = number of patients with characteristic within group. % = # with defined risk factor/# with HM. Abbreviations: HM = Hematologic malignancy AML = Acute myeloid leukemia; ALL = Acute lymphocytic leukemia; CML = Chronic myeloid leukemia; CLL = Chronic lymphocytic leukemia; NHL = Non-Hodgkin lymphoma; MM = Multiple myeloma; SCT = Stem cell transplant.

**Table 5:** Multiple risk factors for dissemination.

	% (n ^a^)
**Chemotherapy, SCT**	***N ^b^* = 49**
**yes, yes**	28.57% (14)
**yes, no**	57.14% (28)
**no, yes**	2.04% (1)
**no, no**	12.24% (6)
**Corticosteroid, SCT**	***N* = 39**
**yes, yes**	23.08% (9)
**yes, no**	30.77% (12)
**no, yes**	5.13% (2)
**no, no**	41.03% (16)
**Chemotherapy, Corticosteroid**	***N* = 52**
**yes, yes**	53.85% (28)
**yes, no**	30.77% (16)
**no, yes**	7.69% (4)
**no, no**	7.69% (4)

^a^ N = total number of patients with characteristic. ^b^ n = number of patients with defined characteristics within group. % = # with defined characteristic/# total number of patients with characteristic. Abbreviations: SCT = stem cell transplant.

**Table 6:** Traditional risk factors with or without antifungal prophylaxis.

	% (n ^a^)
**chemotherapy, anti-fungal prophylaxis**	***N ^b^* = 46**
**yes, yes**	45.65% (21)
**yes, no**	39.13% (18)
**no, yes**	2.17% (1)
**no, no**	13.04% (6)
**corticosteroid, anti-fungal prophylaxis**	***N* = 39**
**yes, yes**	25.64% (10)
**yes, no**	28.21% (11)
**no, yes**	15.38% (6)
**no, no**	30.77% (12)
**SCT, anti-fungal prophylaxis**	***N* = 44**
**yes, yes**	27.27% (12)
**yes, no**	9.09% (4)
**no, yes**	22.73% (10)
**no, no**	40.91% (18)

^a^ N = total number of patients with characteristic. ^b^ n = number of patients with defined characteristics within the group. % = # with defined characteristic/# total number of patients with characteristic. Abbreviations: SCT = stem cell transplant.

**Table 7:** Antifungal therapy.

	% (n ^a^)
**Antifungal therapy** (*N ^b^* = 69)	91.30% (63)
**Type of therapy** (*N* = 61)	
**Mono therapy**	31.15% (19)
**Multiple therapy**	68.84% (42)
**Regimen** (*N* = 61)	
**Included AmB**	80.33% (49)
***DAmB***	75.51% (37)
***L-AmB***	24.49% (12)
**Included Voriconazole**	50.82% (31)
**Included Caspofungin**	18.03% (11)
**Included Itraconazole**	16.39% (10)
**Included Flucytosine**	13.11% (8)
**Included Posaconazole**	6.55% (4)
**Voriconazole + AmB^c^**	24.59% (15)
**Voriconazole + Caspofungin^c^**	4.92% (3)
**Voriconazole + AmB + Caspofungin^c^**	9.84% (6)
**Other**	11.48% (7)
**Type not disclosed**	7.35% (5)
**Surgery** (*N* = 58)	65.38% (38)

^a^ n = number of patients with defined characteristics within the group. ^b^ N = total number of patients with available data for characteristic. % = # with defined characteristic/# with available data for characteristic. ^c^ Patients may have received an additional regimen to the combination. Abbreviations: AmB = Amphotericin B; DAmB = Deoxycholate Amphotericin B; L-AmB = Liposomal Amphotericin B.

**Table 8:** *Aspergillus* species * identity and related mortality.

	Patients (*N ^a^* = 39)	*Survived*	*Died*
	% (n ^b^)		
***Aspergillus fumigatus***	64.10% (25)	60.00% (15)	40.00% (10)
***A. fumigatus + A. flavus* ^c^**	2.56% (1)	100% (1)	
***A. fumigatus + A. niger* ^d^**	2.56% (1)		100% (1)
***Aspergillus flavus***	25.64% (10)	10.00% (1)	90.00% (9)
***Aspergillus terreus***	2.56% (1)		100% (1)
***Aspergillus felis***	2.56% (1)	100% (1)	

^a^ N = total number of patients with available data for characteristic. ^b^ n = number of patients with defined characteristic within group. % = # with defined characteristic/# total number of patients with characteristic. ^c^ 1 patient positive for *Aspergillus fumigatus* and *flavus* ^d^ 1 patient positive for *Aspergillus fumigatus* and *niger*. * *n* = 37 species not identified. Abbreviations: *A.* = *Aspergillus.*

**Table 9:** Potential factors associated with mortality.

	% Mortality (n ^a^)
**Overall mortality**	53.95% (41)
**Age**	
**<18 years** (*N ^b^ =* 25)	36.00% (9)
**≥18, <50 years** (*N =* 20)	70.00% (14)
**≥50 years** (*N =* 39)	53.57% (15)
**Mortality rate according to HM**	
**AML** (*N* = 30)	50.00% (15)
**ALL** (*N* = 25)	48.00% (12)
**CML** (*N* = 1)	100.00% (1)
**CLL** (*N* = 8)	25.00% (2)
**NHL** (*N* = 11)	90.91% (10)
**MM** (*N* = 1)	100.00% (1)
**Mortality rate according to chemotherapy at time of IPA diagnosis**	
**Chemotherapy** (*N* = 59)	59.32% (35)
**No** (*N* = 8)	25.00% (2)
**Mortality rate according to SCT prior to IPA diagnosis**	
**SCT** (*N* = 21)	76.19% (16)
**No** (*N* = 37)	35.14% (13)
**Mortality rate according to corticosteroids at time of IPA diagnosis**	
**Steroids** (*N* = 33)	60.61% (20)
**No** (*N* = 21)	33.33% (7)
**Mortality rate according to prophylactic anti-fungal**	
**Anti-fungal prophylaxis** (*N* = 23)	73.91% (17)
**No** (*N* = 25)	28.00% (7)
**Mortality rate according to therapeutic anti-fungal**	
**Anti-fungal therapy** (*N* = 66)	51.52% (34)
**No** (*N* = 3)	100.00% (3)
**Mortality rate according to surgical intervention post-diagnosis**	
**Surgical intervention** (*N* = 26)	34.62% (9)
**No** (*N =* 32)	62.50% (20)
**Mortality rate according to therapeutic anti-fungal & surgical intervention**	
**Surgical intervention, anti-fungal therapy** (*N* = 25)	36.00% (9)
**No Surgical intervention, anti-fungal therapy** (*N* = 26)	61.54% (16)

^a^ n = number of patients died within characteristic group. ^b^ N = total number of patients with characteristic. % = # died within characteristic group/# total number of patients with characteristic. Abbreviations: HM = Hematologic malignancy AML = Acute myeloid leukemia; ALL = Acute lymphocytic leukemia; CML = Chronic myeloid leukemia; CLL = Chronic lymphocytic leukemia; NHL = Non-Hodgkin’s lymphoma; MM = Multiple myeloma; SCT = Stem cell transplant.

## Data Availability

All data analyzed during this study are included in this article. Further inquiries can be directed to the corresponding author.

## Supplementary Materials

The following supporting information can be downloaded at: https://www.mdpi.com/article/10.3390/jof8070722/s1, Scheme S1. Overall synthesis of quality assessment and risk of bias; Table S1: Literature search results, Table S2: Quality assessment for each article included in the systematic review and overall level of bias, Table S3: Data for HM, neutropenia status, inclusion of chemotherapy, and outcome of individual patients from included studies.
