# Supplementary material for: A Systematic Review to Assess the Relationship between Disseminated Cerebral Aspergillosis, Leukemias and Lymphomas, and Their Respective Therapeutics
Source: J Fungi (Basel). 2022 Jul 11;8(7):722. doi: 10.3390/jof8070722 (PMC9320744; doi:10.3390/jof8070722)
Supplement: Supplementary file 1 [file jof-08-00722-s001.zip › jof-1768579-supplementary.pdf]

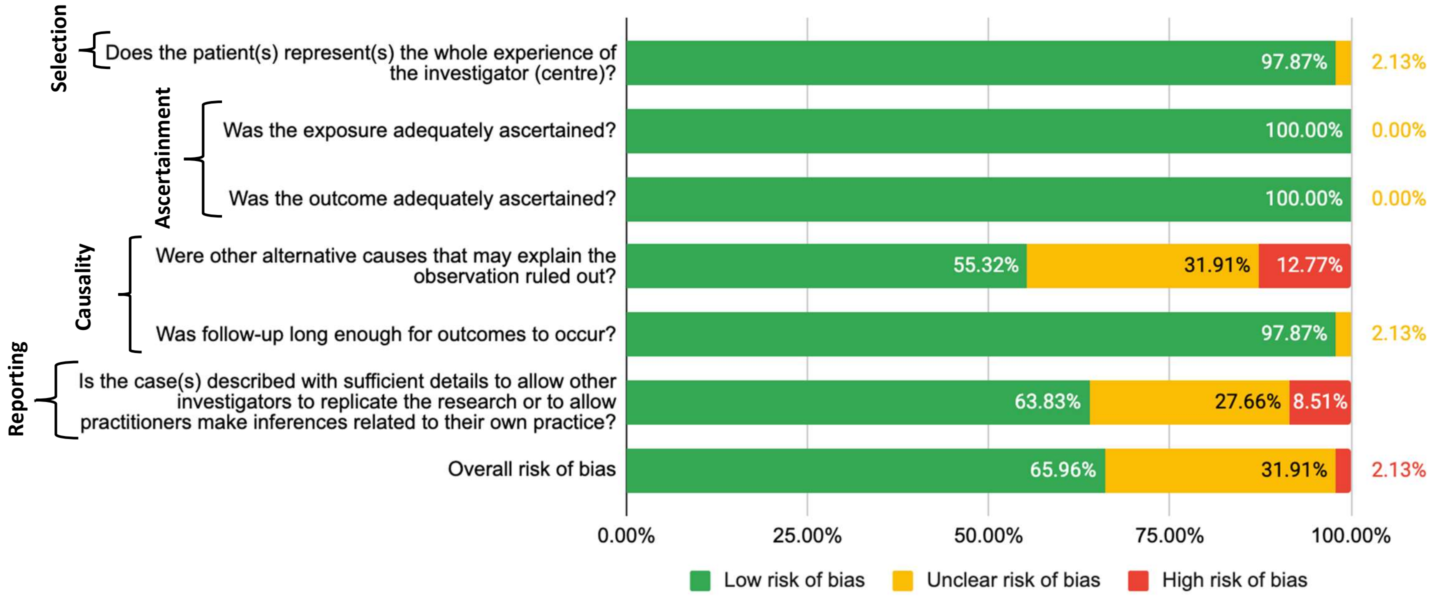

Scheme S1. Overall synthesis of quality assessment and risk of bias.

Supplementary Table S1. Literature search results

Date of Search	Database	Search String	Total results retrieved	Search Details
**18-May-21**	PubMed	(("Aspergillosis"[Mesh] AND disseminat*[tw]) OR (disseminat*[tw] AND aspergillosis[tw]) OR "Neuroaspergillosis"[Mesh] OR "neuroaspergillosis"[tw] OR "nervous system invasive aspergillosis"[tw] OR "cerebral aspergillosis"[tw] OR "central nervous system aspergillosis"[tw] OR "CNS aspergillosis"[tw] OR "brain aspergillosis"[tw] OR "intracranial aspergillosis"[tw] OR "cranial aspergillosis"[tw]) AND ("Antineoplastic Agents"[Mesh] OR "Antineoplastic"[tw] OR "Antineoplastic Agent"[tw] OR "Antineoplastic Agents"[tw] OR "Antineoplastic Drug"[tw] OR "Antineoplastic Drugs"[tw] OR Antineoplastic[tw] OR "Antitumor Drug"[tw] OR "Antitumor Drugs "[tw] OR "Antitumor Agent"[tw] OR "Antitumor Agents"[tw] OR "Cancer Chemotherapy Drug"[tw] OR "Cancer Chemotherapy Drugs"[tw] OR "Cancer Chemotherapy Agent"[tw] OR "Cancer Chemotherapy Agents"[tw] OR "Chemotherapy, Cancer, Regional Perfusion"[Mesh] OR "Neoplasms/drug therapy"[Mesh] OR "Neoplasms/administration and dosage"[Mesh] OR ((cancer*[tw] OR neoplasm*[tw] OR "hematologic malignancy"[tw] OR "hematologic malignancies"[tw] OR leukemia[tw] OR lymphoma[tw]) AND (chemotherapy[tw] OR antineoplastic[tw] OR antitumor[tw] OR antitumour[tw] OR anti-angiogenic[tw] OR immunomodulary[tw] OR cytotoxic[tw] OR chemotherapeutic*[tw] OR immunomodulator*[tw] OR immunosuppressant*[tw] OR immunocompromis*[tw])))	213	
**18-May-21**	Embase	((disseminated NEAR/5 aspergillosis) OR (dissemination NEAR/5 aspergillosis) OR 'central nervous system aspergillosis'/exp OR 'central nervous system aspergillosis' OR ‘neuroaspergillosis’ OR ‘nervous system invasive aspergillosis’ OR ‘cerebral aspergillosis’ OR ‘CNS aspergillosis’ OR ‘brain aspergillosis’ OR ‘intracranial aspergillosis’ OR ‘cranial aspergillosis’) AND ('cancer chemotherapy'/exp OR (cancer NEAR/5 chemotherapy) OR 'antineoplastic agent'/exp OR 'antineoplastic agent' OR ((‘neoplasm’ OR ‘hematologic malignancy’ OR ‘leukemia’ OR ‘lymphoma’) AND (‘chemotherapy’ OR ‘antineoplastic’ OR ‘antitumor’ OR ‘antitumour’ OR ‘anti-angiogenic’ OR ‘immunomodulary’ OR ‘cytotoxic’ OR ‘chemotherapeutic’ OR ‘immunomodulator’ OR ‘immunosuppressant’ OR ‘immunocompromised’))) AND 'article'/it AND 'human'/de	356	All fields

**18-May-21**	CINAHL Plus with Full Text	((disseminated N5 aspergillosis) OR (dissemination N5 aspergillosis) OR central nervous system aspergillosis OR central nervous system aspergillosis OR neuroaspergillosis OR nervous system invasive aspergillosis OR cerebral aspergillosis OR CNS aspergillosis OR brain aspergillosis OR intracranial aspergillosis OR cranial aspergillosis) AND (cancer chemotherapy OR (cancer N5 chemotherapy) OR antineoplastic agent OR ((neoplasm OR hematologic malignancy OR leukemia OR lymphoma) AND (chemotherapy OR antineoplastic OR antitumor OR antitumour OR anti-angiogenic OR immunomodulary OR cytotoxic OR chemotherapeutic OR immunomodulator OR immunosuppressant OR immunocompromised)))	18	All fields
**18-May-21**	Web of Science - Science Index Expanded	((disseminated NEAR aspergillosis) OR (dissemination NEAR aspergillosis) OR central nervous system aspergillosis OR central nervous system aspergillosis OR neuroaspergillosis OR nervous system invasive aspergillosis OR cerebral aspergillosis OR CNS aspergillosis OR brain aspergillosis OR intracranial aspergillosis OR cranial aspergillosis) AND (cancer chemotherapy OR (cancer NEAR chemotherapy) OR antineoplastic agent OR ((neoplasm OR hematologic malignancy OR leukemia OR lymphoma) AND (chemotherapy OR antineoplastic OR antitumor OR antitumour OR anti-angiogenic OR immunomodulary OR cytotoxic OR chemotherapeutic OR immunomodulator OR immunosuppressant OR immunocompromised)))	156	Topic
**18-May-21**	www.greylit.org	((disseminated aspergillosis) OR (dissemination aspergillosis) OR central nervous system aspergillosis OR central nervous system aspergillosis OR neuroaspergillosis OR nervous system invasive aspergillosis OR cerebral aspergillosis OR CNS aspergillosis OR brain aspergillosis OR intracranial aspergillosis OR cranial aspergillosis) AND (cancer chemotherapy OR (cancer chemotherapy) OR antineoplastic agent OR ((neoplasm OR hematologic malignancy OR leukemia OR lymphoma) AND (chemotherapy OR antineoplastic OR antitumor OR antitumour OR anti-angiogenic OR immunomodulary OR cytotoxic OR chemotherapeutic OR immunomodulator OR immunosuppressant OR immunocompromised)))	0	

Supplementary Table S2. Quality assessment for each article used in the systematic review and overall level of bias.

Study ID	Does the patient(s) represent(s) the whole experience of the investigator (centre)?	Was the exposure adequately ascertained?	Was the outcome adequately ascertained?	Were other alternative causes that may explain the observation ruled out?	Was follow-up long enough for outcomes to occur?	Is the case(s) described with sufficient details to allow other investigators to replicate the research?	Overall level of Bias
**Zwitserloot 2008**	Yes	Yes	Yes	Yes	Yes	Yes	Low
**Yeh 2007**	Yes	Yes	Yes	Yes	Yes	Unclear	Low
**Wright 2003**	Yes	Yes	Yes	Yes	Yes	Unclear	Low
**Wandroo 2006**	Yes	Yes	Yes	Yes	Yes	Yes	Low
**Walsh 1985**	Yes	Yes	Yes	Yes	Yes	Unclear	Low
**vanderLinden 2011**	Yes	Yes	Yes	Yes	Yes	Unclear	Low
**Trigg 1993**	Yes	Yes	Yes	Yes	Yes	Yes	Low
**Tracy 1983**	Yes	Yes	Yes	Yes	Yes	Yes	Low
**Tattevin 2004**	Yes	Yes	Yes	Unclear	Yes	Unclear	Unclear
**Sparano 1992**	Yes	Yes	Yes	Yes	Yes	Unclear	Low
**Schauwvlieghe 2020**	Yes	Yes	Yes	Yes	Unclear	Yes	Low
**SchamrothPravda 2019**	Yes	Yes	Yes	Yes	Yes	Unclear	Low
**Sancho 1997**	Yes	Yes	Yes	Yes	Yes	Yes	Low
**Sakata 2021**	Yes	Yes	Yes	Unclear	Yes	Yes	Low
**Ruchlemer 2019**	Yes	Yes	Yes	Unclear	Yes	Unclear	Unclear
**Prakash 2012**	Yes	Yes	Yes	Yes	Yes	Yes	Low
**Pongbhaesaj 2004**	Yes	Yes	Yes	Unclear	Yes	Unclear	Unclear
**Peng 2015**	Yes	Yes	Yes	Unclear	Yes	Yes	Low
**Pascale 2015**	Yes	Yes	Yes	Unclear	Yes	Yes	Low
**Palmisani 2017**	Yes	Yes	Yes	No	Yes	Yes	Unclear
**Nov 1984**	Yes	Yes	Yes	Yes	Yes	Yes	Low
**Mori 1998**	Yes	Yes	Yes	Yes	Yes	Yes	Low

**Middelhof 2005**	Yes	Yes	Yes	Yes	Yes	Yes	Low
**Marbello 2003**	Yes	Yes	Yes	Yes	Yes	Yes	Low
**Mahlknecht 1997**	Yes	Yes	Yes	Unclear	Yes	Yes	Low
**Lionakis 2017**	Yes	Yes	Yes	Unclear	Yes	Yes	Low
**Kurdow 2005**	Yes	Yes	Yes	Yes	Yes	Yes	Low
**Kreisel 1991**	Yes	Yes	Yes	Yes	Yes	No	Unclear
**Kawanami 2002**	Yes	Yes	Yes	Yes	Yes	Unclear	Low
**Kaste 2000**	Yes	Yes	Yes	Unclear	Yes	Unclear	Unclear
**Iwen 1997**	Yes	Yes	Yes	Unclear	Yes	Unclear	Unclear
**Iwen 1993**	Yes	Yes	Yes	Unclear	Yes	Unclear	Unclear
**Im 2012**	Yes	Yes	Yes	No	Yes	Yes	Unclear
**Hummel 2006**	Unclear	Yes	Yes	Unclear	Yes	No	High
**Henze 1982**	Yes	Yes	Yes	No	Yes	Yes	Unclear
**Guermazi 2002**	Yes	Yes	Yes	No	Yes	Yes	Unclear
**Groll 1999**	Yes	Yes	Yes	Unclear	Yes	Yes	Low
**Gaye 2018**	Yes	Yes	Yes	Yes	Yes	Yes	Low
**Flatt 2012**	Yes	Yes	Yes	No	Yes	Yes	Unclear
**Faisal 2019**	Yes	Yes	Yes	Yes	Yes	No	Unclear
**Eichenberger 2020**	Yes	Yes	Yes	Yes	Yes	No	Unclear
**DeLeonardis 2020**	Yes	Yes	Yes	No	Yes	Yes	Unclear
**Damaj 2004**	Yes	Yes	Yes	Yes	Yes	Yes	Low
**Beresford 2019**	Yes	Yes	Yes	Yes	Yes	Yes	Low
**Athanassiadou 2005**	Yes	Yes	Yes	Unclear	Yes	Yes	Low
**Anciones 2018**	Yes	Yes	Yes	Unclear	Yes	Yes	Low
**Amanati 2020**	Yes	Yes	Yes	Yes	Yes	Yes	Low

Low bias = 1 or fewer questions with the answer of "unclear," Unclear bias = 2 questions with the answer of "unclear" or 1 with the answer of "no," high risk of bias = 3 or more questions with the answer of "unclear" or 2 questions with the answer of "unclear" and 1 with the answer of "no" or 2 questions with the answer of "no"

Supplementary Table S3. Data for HM, neutropenia status, inclusion of chemotherapy, and outcome of individual patients from included studies**.**

Patient #	Underlying Disease	Reported ANC or WBC Status (cells/ul)	Chemotherapy	Outcome
**1**	CLL	<100	yes	Survived
**2**	CLL	7100	no	Survived
**3**	ALL	≤500	yes	Survived
**4**	ALL	≤500	yes	Died
**5**	AML	NA	NA	Died
**6**	AML	NA	yes	Died
**7**	MM	Neutropenic	yes	Died
**8**	ALL	NA	NA	Survived
**9**	AML	NA	NA	Died
**10**	ALL	NA	NA	Survived
**11**	NHL	>550	yes	Died
**12**	NHL	≤500	yes	Died
**13**	NHL	≤500	yes	Died
**14**	NHL	<500	yes	Died
**15**	NHL	<500	yes	Died
**16**	ALL	<500	yes	Died
**17**	ALL	NA	NA	Died
**18**	AML	NA	NA	Survived
**19**	AML	<100	yes	Died
**20**	AML	<100	yes	Died
**21**	ALL	<100	yes	Died
**22**	ALL	Granulocytopenia	yes	Survived
**23**	AML	Granulocytopenia	yes	Died
**24**	NHL	NA	yes	Died
**25**	NHL	Not neutropenic	yes	Died
**26**	NHL	<500	yes	Survived
**27**	AML	NA	yes	Survived
**28**	AML	NA	yes	Survived
**29**	AML	NA	yes	Died
**30**	AML	NA	yes	Survived
**31**	CLL	NA	yes	Died
**32**	CLL	Not neutropenic	no	Died
**33**	ALL	NA	yes	Survived
**34**	ALL	NA	yes	Survived
**35**	AML	<100	yes	Died
**36**	ALL	<100	yes	Died
**37**	AML	<100	yes	Died
**38**	ALL	<100	yes	Died
**39**	AML	<100	yes	Died

**40**	ALL	<100	yes	Died
**41**	NHL	NA	NA	Died
**42**	AML	NA	NA	Survived
**43**	AML	1,200^	yes	Survived
**44**	AML	10,200^	no	Survived
**45**	NHL	650^	yes	Died
**46**	ALL	220^	yes	Died
**47**	AML	<500	yes	Died
**48**	AML	NA	yes	Survived
**49**	ALL	480	yes	Survived
**50**	AML	100	yes	Survived
**51**	ALL	NA	yes	Survived
**52**	AML	1100^	yes	Died
**53**	AML	NA	yes	Died
**54**	NHL	1500	yes	Died
**55**	ALL	4500^ (38% neutrophils)	yes	Died
**56**	AML	3393	no	Died
**57**	ALL	3900^	yes	Died
**58**	ALL	NA	yes	Died
**59**	AML	NA	no	Survived
**60**	AML	NA	yes	Survived
**61**	AML	Neutropenic	yes	Died
**62**	CLL	<100	yes	Survived
**63**	AML	<500	yes	Survived
**64**	AML	Neutropenic	yes	Survived
**65**	AML	2,420^	NA	Survived
**66**	ALL	NA	yes	Survived
**67**	CML	Neutropenic	yes	Died
**68**	ALL	<500	yes	Survived
**69**	CLL	1000	no	Survived
**70**	CLL	3200^	no	Survived
**71**	ALL	Neutropenic	yes	Survived
**72**	AML	Neutropenic	yes	Survived
**73**	CLL	2600	no	Survived
**74**	ALL	Neutropenic	yes	Survived
**75**	ALL	Neutropenic	yes	Died
**76**	ALL	NA	yes	Survived

^ = White blood cell count

Abbreviations: ANC = Absolute neutrophil count; WBC = White blood cell; NA = Data not available; AML = Acute myeloid leukemia; ALL = Acute lymphocytic leukemia; CML = Chronic myeloid leukemia; CLL = Chronic lymphocytic leukemia; NHL = non-Hodgkin’s lymphoma; MM = Multiple myeloma
